# Identification of steroid-induced osteonecrosis of the femoral head biomarkers based on immunization and animal experiments

**DOI:** 10.1186/s12891-024-07707-4

**Published:** 2024-07-29

**Authors:** Dongqiang Luo, Xiaolu Gao, Xianqiong Zhu, Jiayu Wu, Qingyi Yang, Ying Xu, Yuxuan Huang, Xiaolin He, Yan Li, Pengfei Gao

**Affiliations:** 1 Nanfang College Guangzhou, Guangzhou, 510970 China; 2 Guangzhou University of Chinese Medicine, Guangzhou, 510006 China; 3 Guangdong Pharmaceutical University, Guangzhou, 510006 China; 4 Clifford Hospital, Guangzhou, 511496 China

**Keywords:** Steroid-induced, Osteonecrosis, Femoral head, Immunization, Animal experiments

## Abstract

**Background:**

Steroid-induced osteonecrosis of femoral head (SONFH) is a severe health risk, and this study aims to identify immune-related biomarkers and pathways associated with the disease through bioinformatics analysis and animal experiments.

**Method:**

Using SONFH-related datasets obtained from the GEO database, we performed differential expression analysis and weighted gene co-expression network analysis (WGCNA) to extract SONFH-related genes. A protein-protein interaction (PPI) network was then constructed, and core sub-network genes were identified. Immune cell infiltration and clustering analysis of SONFH samples were performed to assess differences in immune cell populations. WGCNA analysis was used to identify module genes associated with immune cells, and hub genes were identified using machine learning. Internal and external validation along with animal experiments were conducted to confirm the differential expression of hub genes and infiltration of immune cells in SONFH.

**Results:**

Differential expression analysis revealed 502 DEGs. WGCNA analysis identified a blue module closely related to SONFH, containing 1928 module genes. Intersection analysis between DEGs and blue module genes resulted in 453 intersecting genes. The PPI network and MCODE module identified 15 key targets enriched in various signaling pathways. Analysis of immune cell infiltration showed statistically significant differences in CD8 + t cells, monocytes, macrophages M2 and neutrophils between SONFH and control samples. Unsupervised clustering classified SONFH samples into two clusters (C1 and C2), which also exhibited significant differences in immune cell infiltration. The hub genes (ICAM1, NR3C1, and IKBKB) were further identified using WGCNA and machine learning analysis. Based on these hub genes, a clinical prediction model was constructed and validated internally and externally. Animal experiments confirmed the upregulation of hub genes in SONFH, with an associated increase in immune cell infiltration.

**Conclusion:**

This study identified ICAM1, NR3C1, and IKBKB as potential immune-related biomarkers involved in immune cell infiltration of CD8 + t cells, monocytes, macrophages M2, neutrophils and other immune cells in the pathogenesis of SONFH. These biomarkers act through modulation of the chemokine signaling pathway, Toll-like receptor signaling pathway, and other pathways. These findings provide valuable insights into the disease mechanism of SONFH and may aid in future drug development efforts.

**Supplementary Information:**

The online version contains supplementary material available at 10.1186/s12891-024-07707-4.

## Introduction

Glucocorticoids are frequently employed in the therapeutic management of inflammatory and connective tissue diseases, including systemic lupus erythematosus, Sjogren’s syndrome, rheumatoid arthritis, and severe acute respiratory syndrome [1, 2]. A prevalent complication arising from glucocorticoid treatment is steroid-induced osteonecrosis of the femoral head (SONFH). Studies from China and Japan underscore glucocorticoids as a significant risk factor for nontraumatic SONFH [3, 4]. Without intervention, SONFH typically progresses, leading to hip joint degeneration and compromised function, significantly reducing patients’ quality of life. Annually, an estimated 20,000 to 30,000 new cases of SONFH are recorded worldwide, not taking into account transmission [5], and this figure is expected to rise due to the COVID-19 pandemic [6]. Over 50% of affected individuals ultimately require joint replacement surgery. Despite this, the pathogenesis of SONFH still needs to be fully elucidated. The detection of SONFH in its early stages remains elusive, owing to the absence of adequate diagnostic methods, even with traditional imaging techniques [7]. Moreover, the accessibility and economic demands of these imaging methods pose additional challenges, particularly in regions like China [8, 9]. While total hip arthroplasty substantially enhances patients’ quality of life, the associated financial and psychological impacts are considerable for many [10]. Hence, there is a critical need to understand the molecular and biological underpinnings of SONFH and to identify new biomarkers to facilitate early diagnosis and tailor treatment strategies.

**Fig. 1:**
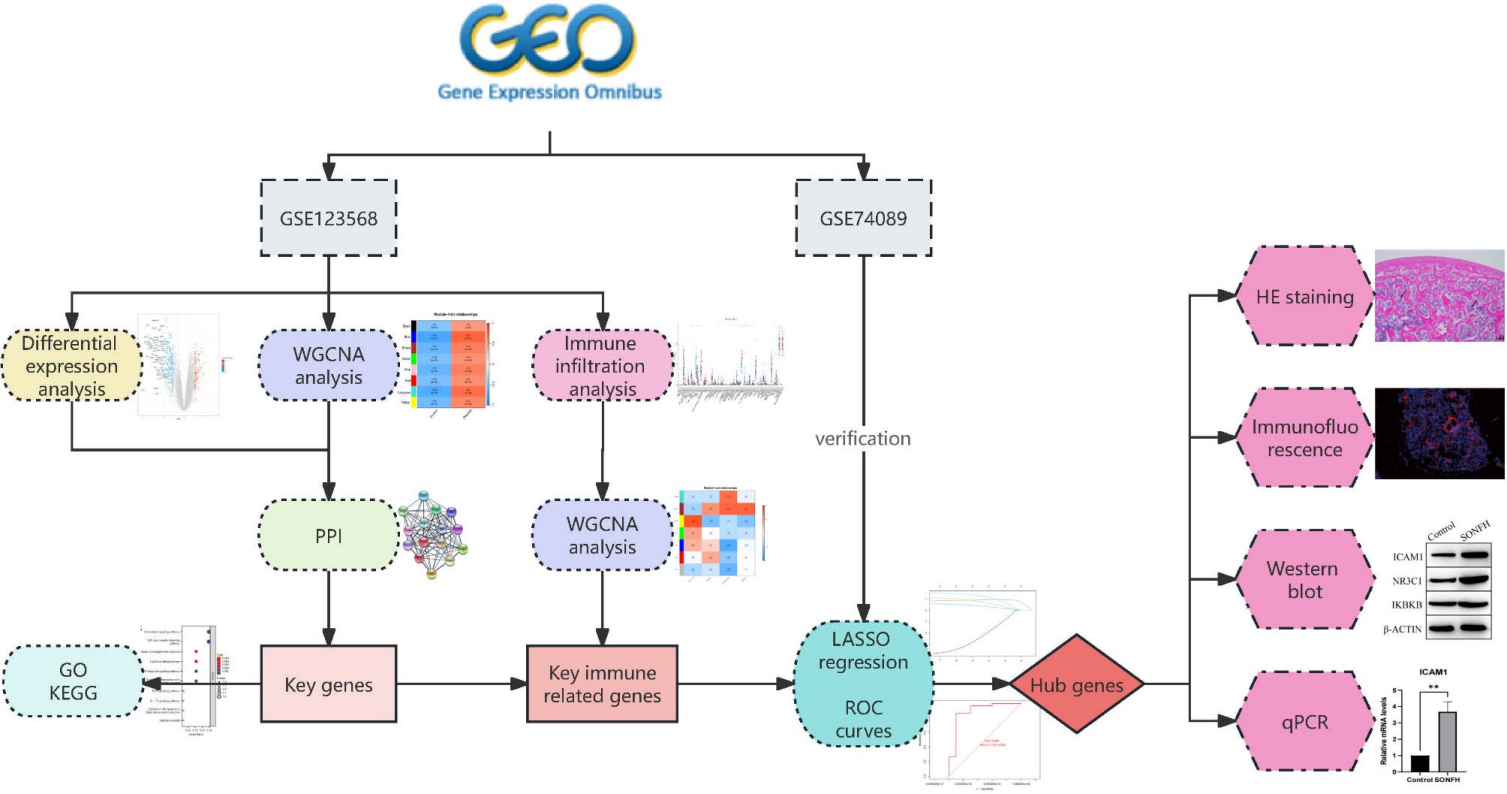
Flow chart

Steroid medications significantly modulate the immune microenvironment. Orally administered steroids exert anti-inflammatory effects by hindering transcription factor activation, diminishing neutrophil adherence to endothelial cells, and inhibiting the production of inflammatory cytokines. Prolonged administration of oral steroids is associated with a notable reduction in CD4 cells and frequently a decrease in serum immunoglobulin G (IgG) levels [11]. The link between SONFH and immune system dysregulation is apparent. Bioinformatics, a cross-disciplinary domain that merges information science with molecular biology, is increasingly employed in gene expression analysis and biomarker identification. Researchers often utilize microarray or high-throughput sequencing to generate extensive data sets accessible through public databases such as ArrayExpress and the Gene Expression Omnibus (GEO). Recently, machine learning and weighted gene co-expression network analysis (WGCNA) have emerged as powerful tools for pinpointing biomarkers intricately associated with diseases [12, 13]. These methodological advances offer promising avenues for enhancing our comprehension of disease-immune system interactions. Consequently, this study is designed to elucidate the mechanistic pathways of SONFH by leveraging WGCNA, machine-learning techniques, Immune infiltration analysis,, and further experiments, as depicted in the accompanying flow chart (Fig. 1).

## Methods and materials

### Data acquisition and preparation

The dataset GSE123568 was sourced from the GEO database (https://www.ncbi.nlm.nih.gov/geo/) using the search term “Steroid-induced Osteonecrosis of the Femoral Head” and is based on the Affymetrix Human Gene Expression Array platform (GPL15207). This dataset contains 40 samples, of which 30 are SONFH samples, and 10 are control samples. For purposes of validation, the GSE74089 dataset, based on the Agilent-026652 Whole Human Genome Microarray 4 × 44 K v2 platform (GPL13497), was also obtained, comprising 8 samples — 4 samples with necrosis of the femoral head and 4 control samples. Following the acquisition of the datasets above, normalization and binarization of the gene expression matrix were performed using the GEOquery [14] package, categorizing the sample disease status into two groups: the diseased group (1) and the control group (0).

### Differential expression analysis

The GSE123568 dataset underwent differential expression analysis via the limma [15] package to identify differentially expressed genes (DEGs). DEGs were screened with |logFC|≥1, *p* < 0.05. And the DEGs were classified according to their expression patterns to the SONFH group and control group, with those showing increased expression in the SONFH group designated as up-regulated and those with decreased expression set as down-regulated.

### Weighted gene co-expression network analysis(WGCNA)

The GSE123568 dataset was analyzed employing the WGCNA package [16] to compute gene correlations. Subsequently, these correlation coefficients were transformed using a weighting function to construct an Adjacency Matrix. A one-step method was applied to select a soft threshold β for assembling a scale-free network. The dynamic hybrid cutting method was then used to detect modules exhibiting significant correlations among their genes. The correlation coefficients between each identified module and the phenotypic traits were calculated. For this study, the disease status of the samples was used as the phenotypic trait, leading to the identification of module genes associated with the disease status. By employing a Venn diagram, we were able to pinpoint the intersection of DEGs with the genes in the most relevant module, thereby identifying the genes associated with SONFH as disease-related genes.

### Protein-protein interaction (PPI) network construction

Disease genes associated with SONFH were extracted and imported into the STRING database [17] (https://cn.string-db.org/). The species was set to “Homo sapiens”, and the minimum required interaction score was set to medium confidence (0.400) to create a protein-protein interaction (PPI) network. Following this, the PPI network was imported into the Cytoscape 3.7.1 software for network topology analysis. To identify the core subnetwork of the network, the MCODE module was used to determine critical subnetworks and targets as key targets.

### Enrichment Analysis

Key genes implicated in SONFH were identified, and enrichment analysis was carried out using the “clusterProfiler” [18] package to assess Gene Ontology (GO) terms and Kyoto Encyclopedia of Genes and Genomes (KEGG) pathways. The significance of the enrichment results was determined by applying a threshold for the adjusted p-value, with a cutoff of *P* < 0.05.

### Differences in immune cell infiltration between SONFH and the control group

The xCell algorithm [19] was employed to examine immune cell infiltration within each sample included in the GSE123568 dataset. The Wilcoxon test was used to evaluate differences in immune cell infiltration between the SONFH and control groups. xCell is a methodology predicated on gene signatures refined by analysing thousands of pure cell types from diverse origins. It incorporates an innovative approach to reduce confounding effects due to correlations among closely related cell types. xCell signatures have been validated through rigorous in-silico simulations and cytometry-based immunophenotyping, affirming their enhanced performance compared to previous methodologies.

### Unsupervised Clusterin

Unsupervised clustering analysis was conducted on SONFH sample data from GSE123568 using the ConsensusClusterPlus [20] package based on key genes, categorizing SONFH samples into clusters. Simultaneously, the xCell algorithm was used to compare differences in immune cell infiltration among the different clusters.

### WGCNA based on immuno-infiltration results

Only the gene expression matrix and immune cell infiltration results for the SONFH group in GSE123568 were extracted. We employed the WGCNA package once more to construct a scale-free network, using the samples’ immune cell infiltration analysis results as phenotypic traits in this study to further screen for modular genes associated with immune cell infiltration.

### Hub gene screening and prediction model construction

With disease status as a binary outcome variable (SONFH = 1, Control = 0), Lasso regression paired with ten-fold cross-validation was used to identify hub genes from the GSE123568 dataset. Following selecting these hub genes, a two-step validation approach was executed. Internal validation within the GSE123568 dataset was initially conducted to assess the model’s robustness. This was followed by external validation on the GSE74089 dataset to evaluate the model’s generalizability. The model’s predictive performance was determined by generating a Receiver Operating Characteristic (ROC) curve and computing the Area Under the Curve (AUC).

### Animals

Twelve 10-week-old male SD rats were purchased from the Animal Experiment Center of Guangzhou University of Chinese Medicine. All experiments involved in this study were approved by the Animal Ethics Committee of Guangzhou University of Chinese Medicine. The experiments were conducted in accordance with the regulations of animal experiments of Guangzhou University of Traditional Chinese Medicine and ARRIVE guidelines.Rats were randomly divided into 2 groups: control group (*n* = 6) and SONFH group (*n* = 6). Rats in the SONFH group were injected with methylprednisolone (60 mg/kg) intramuscularly in the buttocks 7 times/day, and the rats were weighed before each injection. Meanwhile, the control group was injected with an equal amount of saline. All rats were placed in rat cages with free access to water and food. A 12-hour light/dark cycle was provided. At week 6, rat femoral head tissues were obtained for further experiments. The rats were euthanized by intraperitoneal injection of 130 mg/kg pentobarbital.

### HE staining

The femoral head tissue was fixed in paraformaldehyde solution (pH = 7.4) for 48 h and then decalcified in 10% EDTA for one month. The EDTA solution was changed daily. After the bone tissue was softened, it was embedded in paraffin and cut into 5-µm-thick sections for hematoxylin-eosin (HE) staining. Afterward, we observed and evaluated the microstructure of the bone.

### Immunofluorescence

After dewaxing the paraffin sections to water, place the sections in a container filled with EDTA antigen retrieval buffer (PH = 8.0) and heat to perform antigen retrieval. After cooling, place the sections in PBS (PH = 7.4) and shake on a decolorizing shaker for 5 min. Add autofluorescence quencher agent to the sections for 5 min, followed by a rinse under running water for 10 min, and then incubate with BSA (bovine serum albumin) for 30 min. After removing the blocking solution with filter paper, place the sections in a wet box and incubate overnight at 4 °C. After washing and discarding the primary antibody, add the secondary antibody and incubate in the dark at room temperature for 1 h. After washing and discarding the secondary antibody, add 10 µg/mL DAPI staining solution to the sections and incubate in the dark at room temperature for 10 min. After washing again, seal the slides with an anti-fading mounting medium and store in the dark at 4 °C. Observe and collect images using a fluorescence microscope. The DAPI stained nuclei emit blue light under UV excitation.

The antibodies used in the experiment include: Anti-CD8 (abcam company, ab237709), Anti-CD68 (ab283654), Anti-Myeloperoxidase (abcam company, ab45977).

### Real-time qPCR

Total RNA was extracted from femoral tissue using Trizol (Invitrogen) solution and RNA extraction kit (TaKaRa). Reverse transcription was performed using M-MLV reverse transcriptase solution. The reaction conditions were: pre-denaturation at 95 °C for 120 s; denaturation at 95 °C for 15 s; annealing at 60 °C for 30 s; and extension at 72 °C for 40 cycles. β-actin was used as an internal reference.

### Western blot

Femoral tissues were placed in liquid nitrogen and then ground until pulverized. After adding RIPA lysis buffer to extract the total protein from the tissue. Protein concentration was detected using a dicinchoninic acid (BCA) kit. We transferred the protein samples onto polyvinylidene difluoride (PVDF) membranes by SDS-polyacrylamide (SDS-PAGE) gel electrophoresis and sealed them with 5% skim milk for 2 h. Primary antibodies were added and incubated overnight at 4 °C. The next day, after using TBST washing film twice, the secondary antibody was added and incubated at room temperature for 2 h. β-actin was used as the internal reference protein. Finally, use ImageJ to analyze protein bands. The antibodies used in the experiments included Anti-ICAM1 antibody (abcam company, ab282575), Anti-NR3C1 (mlBio company, Ab-226), CHUK/IKBKB Rabbit Polyclonal Antibody (abclonal company, A20203), Anti-beta Actin antibody (abcam company, ab8227).

## Results

### DEGs screening and WGCNA

**Fig. 2:**
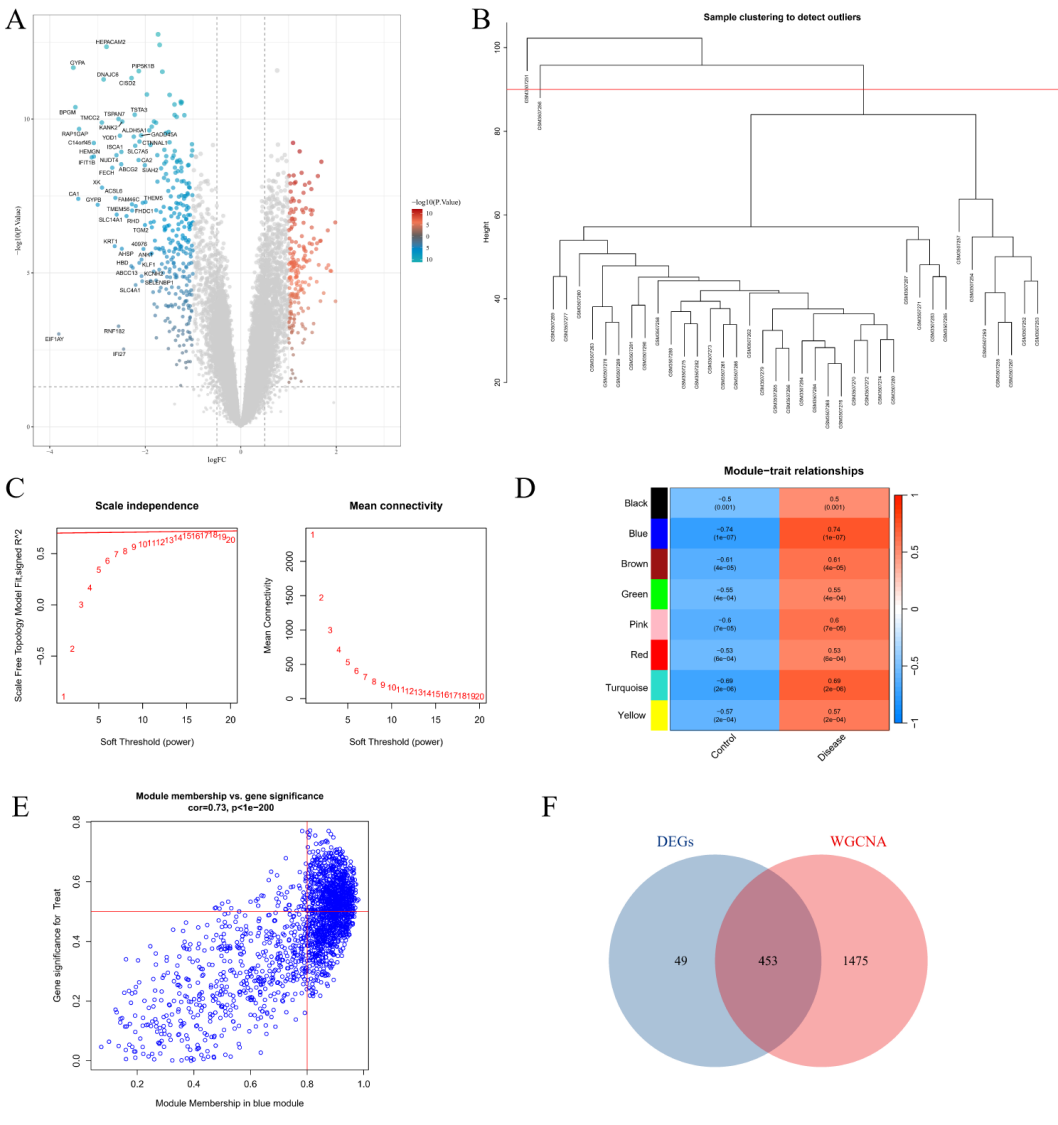
Acquisition of SONFH-Related targets. (**A**) Volcano plot representing differential gene expression analysis. (**B**) Dendrogram depicting sample clustering. (**C**) Screening for the optimal soft-thresholding power. (**D**) WGCNA module detection based on disease presence versus absence. (**E**) Correlation of the blue module with disease status. (**F**) Venn diagram illustrating overlap between DEGs and genes from the WGCNA blue module

Utilizing the limma package, 502 differentially expressed genes (DEGs) were discerned from the GSE123568 dataset. Of these, 197 genes were found to be up-regulated, while 305 were down-regulated (Fig. 2A). Subsequent clustering of the 40 samples in the GSE123568 dataset revealed outlier samples GSM3507251 and GSM3507256 (Fig. 2B), which were subsequently excluded from further analysis. A scale-free network was successfully constructed (Fig. 2C) with the selection of a β value of 18, resulting in a network comprising 8 modules. Notably, the blue module demonstrated the most significant correlation with SONFH (cor = 0.74, *P* = 1e-7) (Fig. 2D and E). From this blue module, 1928 genes were extracted. A Venn diagram was constructed, revealing an intersection of 453 genes between the DEGs and those within the blue module (Fig. 2F).

### Screening SONFH key genes through PPI network and MCODE module

**Fig. 3:**
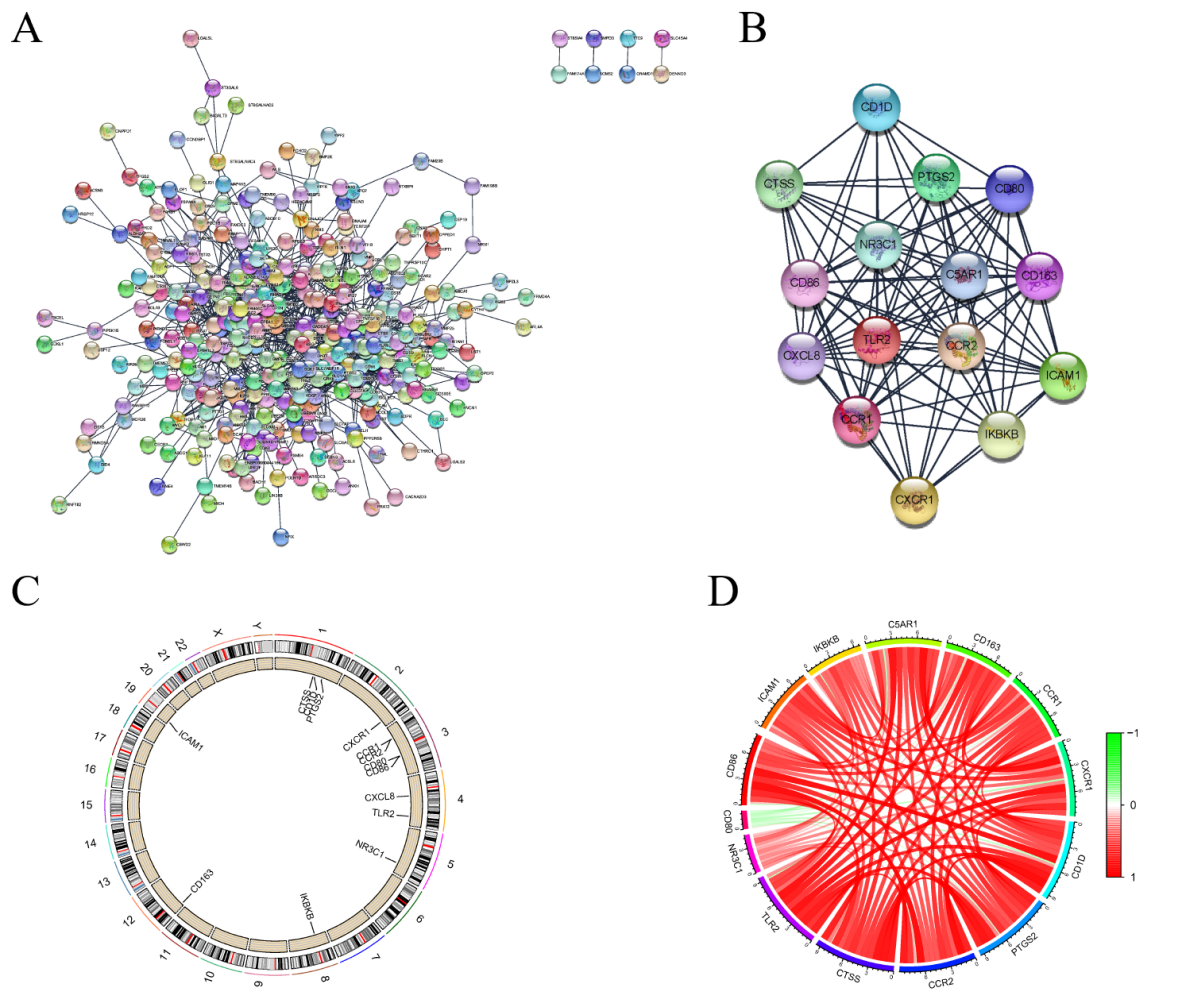
Acquisition of key targets in SONFH. (**A**) Visualization of the protein-protein interaction (PPI) network. (**B**) Identifying the core subnetwork. (**C**) map depicting chromosomal localization of key genes. (**D**) Chord diagram illustrating correlations among key genes

A protein-protein interaction (PPI) network was established by uploading 453 genes that intersect into the STRING database, facilitating the creation of the PPI network visual (Fig. 3A). This network was subsequently analyzed in the Cytoscape software. Through the application of the MCODE algorithm in Cytoscape, the network was segmented into 10 significant clusters. Cluster 1, achieving the highest score, was recognized as the principal cluster within the network (Fig. 3B). It comprises 15 nodes and 92 edges, highlighting key genes implicated in SONFH, such as CD80, CD86, ICAM1, IKBKB, C5AR1, CD163, CXCL8, CCR1, CXCR1, CD1D, PTGS2, CCR2, CTSS, TLR2, and NR3C1, suggesting a genomic interplay associated with SONFH pathogenesis. The analysis of the chromosomal positions for these critical genes uncovered a positive correlation among all genes, with the sole exception of CD80 (Fig. 3C and D).

### Enrichment analysis

**Fig. 4:**
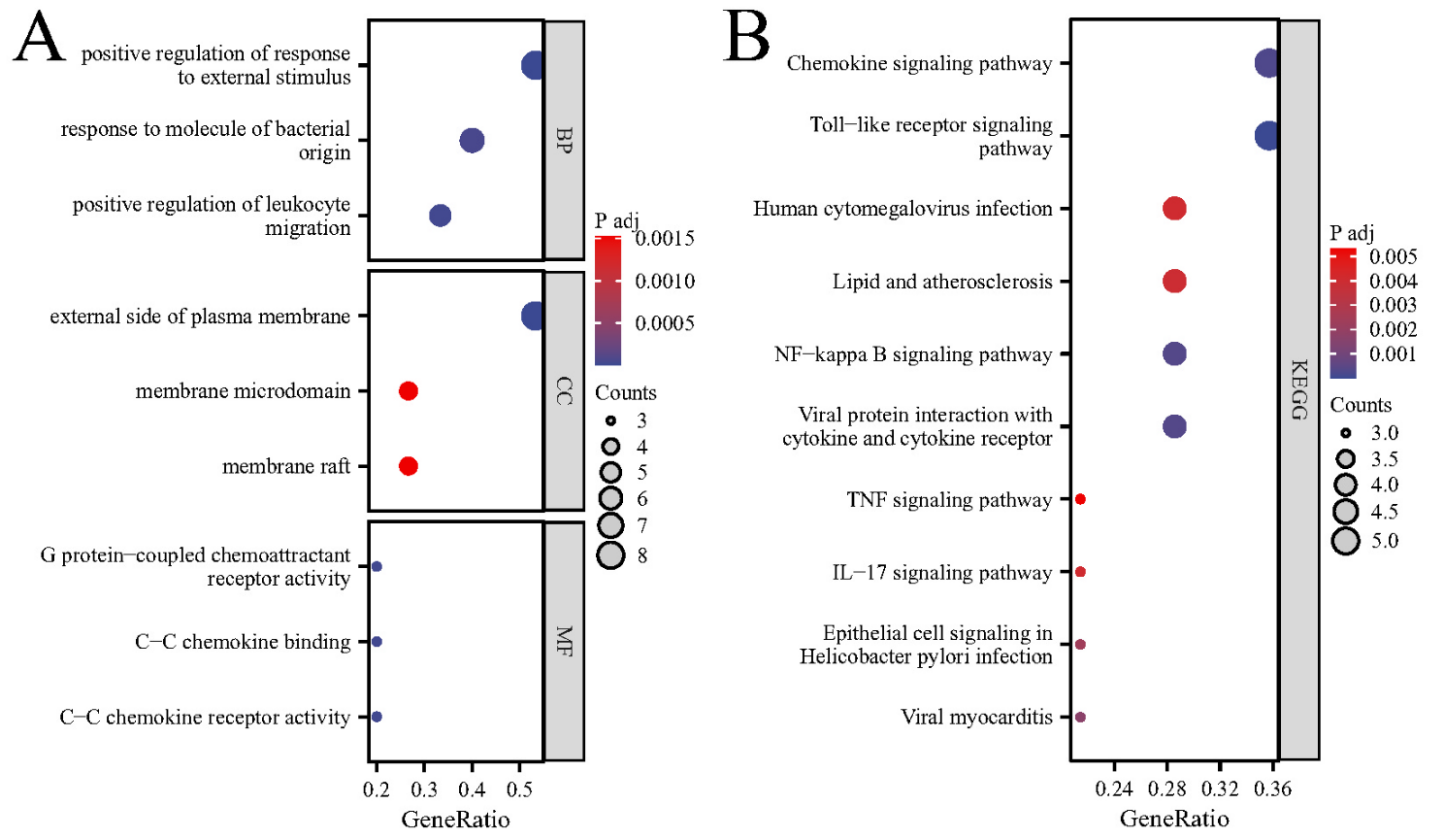
Results of enrichment analysis. (**A**) Results of go enrichment analysis indicating biological processes, cellular components, and molecular functions associated with SONFH. (**B**) Results of KEGG pathway enrichment analysis identifying pathways significantly implicated in SONFH

Applying an adjusted p-value cutoff of *P* < 0.05, GO enrichment analysis indicated that the key genes associated with SONFH predominantly participate in various biological processes, notably the positive regulation of responses to external stimuli, reactions to bacterial molecules, and the positive regulation of leukocyte migration. These SONFH-related key genes are chiefly enriched in cell components such as the external side of the plasma membrane, membrane microdomains, and membrane rafts. In terms of molecular function, there is a significant enrichment in activities related to G protein-coupled chemoattractant receptors, C-C chemokine binding, and C-C chemokine receptor activity (Fig. 4A). The KEGG pathway analysis revealed a primary enrichment of these key SONFH genes in pathways including Chemokine signalling, Toll-like receptor signalling, Lipid and atherosclerosis, NF-kappa B signalling, TNF signalling, and IL-17 signalling (Fig. 4B).

### The difference between the SONFH group and the control group’s immune cell infiltration

**Fig. 5:**
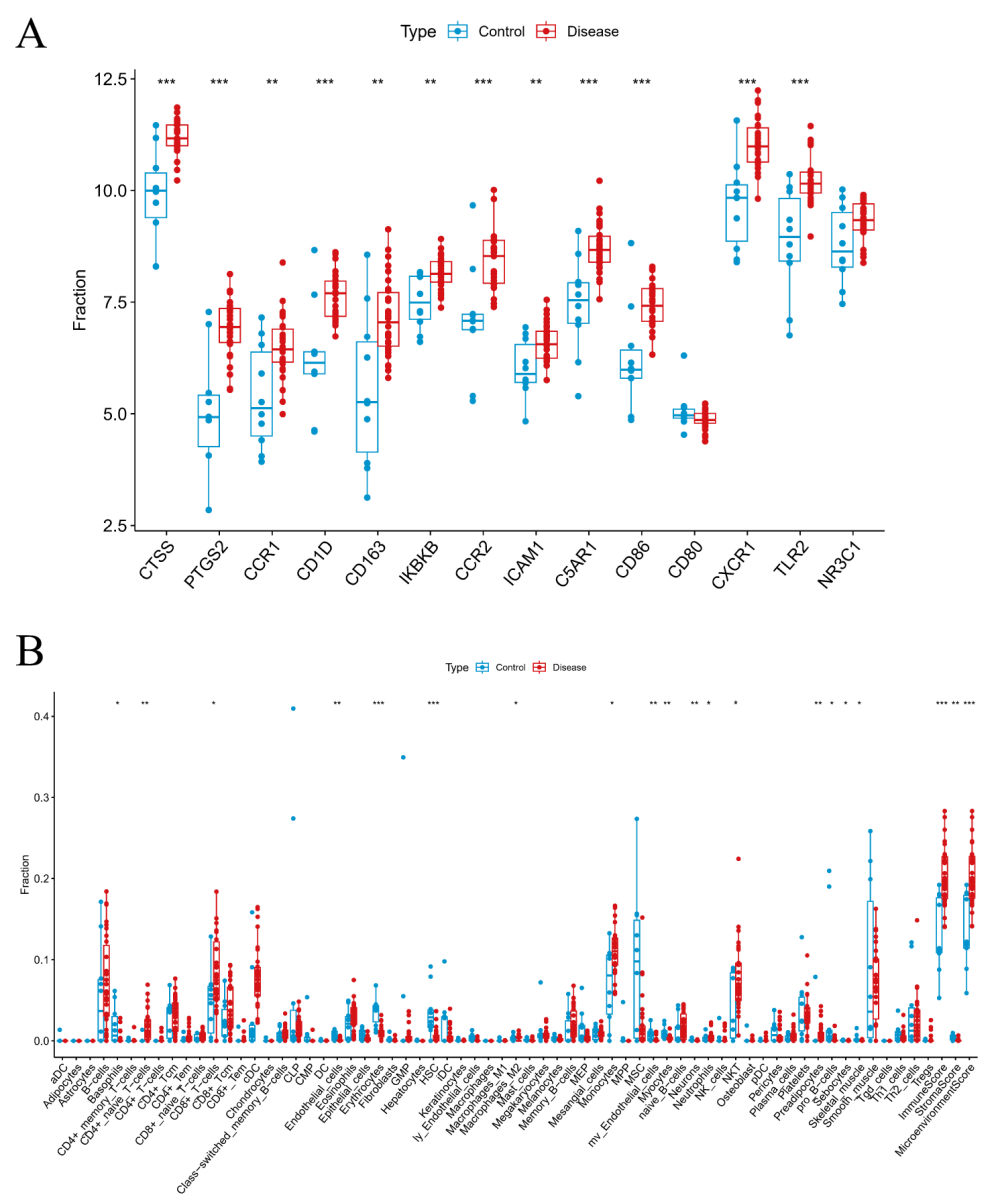
Immune infiltration analysis by disease state. (**A**) Expression differences of key genes between disease and control groups. (**B**) Variations in immune cell infiltration between disease and control groups

The Wilcoxon rank-sum test revealed that the expression levels of key genes in the SONFH group were significantly elevated compared to those in the control group, with the exceptions of CD80 and NR3C1, which did not follow this pattern (*P* < 0.05) (Fig. 5A). Furthermore, an analysis of immune cell infiltration demonstrated notable differences between the SONFH group and the control group, for example, in the level of infiltration of CD8 + t cells, monocytes, macrophages M2 and neutrophils (Fig. 5B).

### Unsupervised clustering and immune cell infiltration analysis

**Fig. 6:**
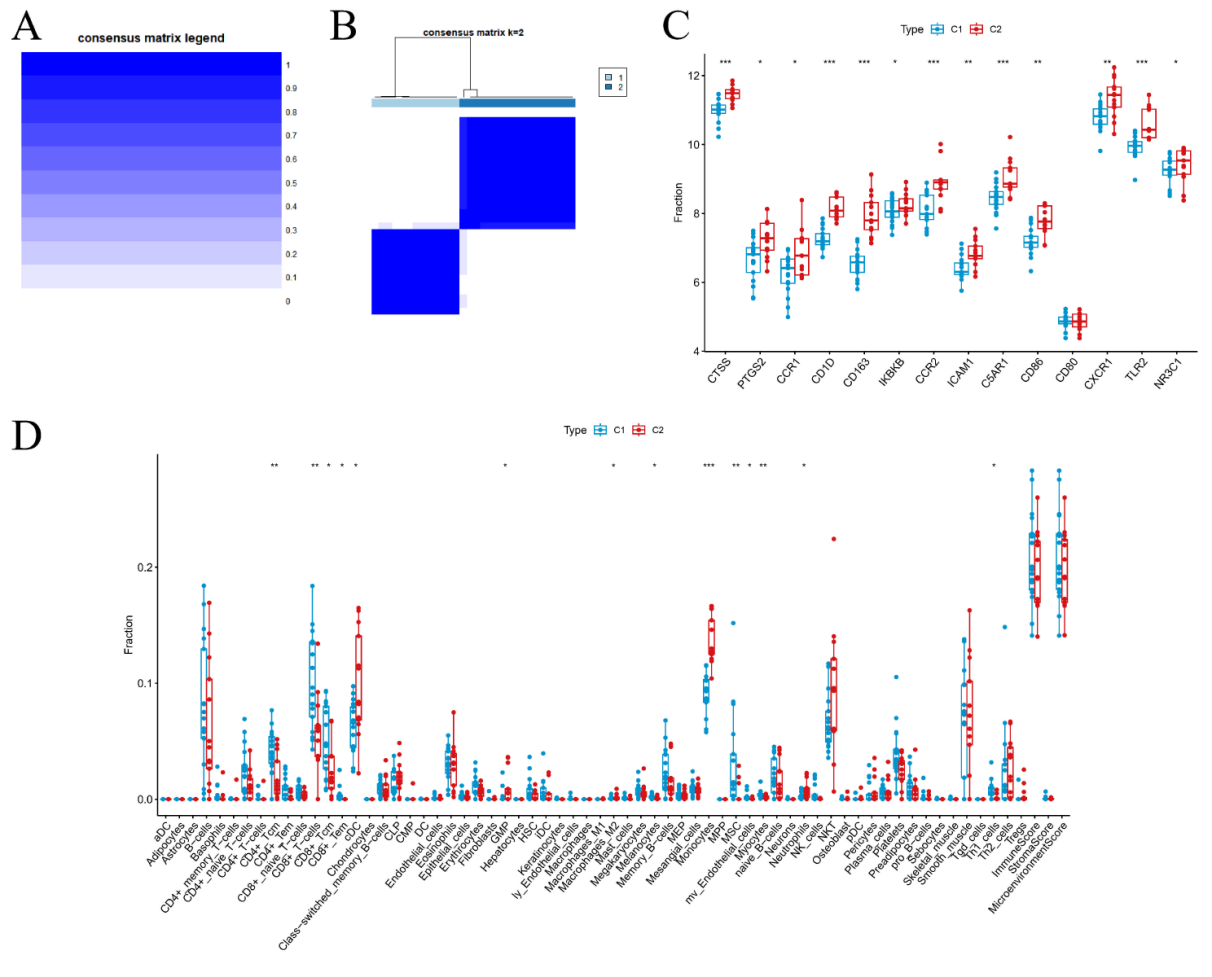
Unsupervised clustering analysis. (**A**) Results of unsupervised clustering. (**B**) Differential expression of key genes across clusters. (**C**) Variations in immune cell infiltration across clusters

To delve deeper into the influence of key genes on immune infiltration in SONFH, we isolated the SONFH sample data for focused examination. Using the ConsensusClusterPlus package, unsupervised clustering analysis of these key genes was conducted. This analysis segregated the SONFH samples into two distinct clusters, labeled C1 and C2 (Fig. 6A and B). Notably, with the exception of CD80, the expression levels of the other key genes were generally higher in the SONFH samples compared to the controls (*P* < 0.05) (Fig. 6C).

Additionally, an analysis of immune cell infiltration within the SONFH samples was performed utilizing the xCell algorithm. The results revealed significant disparities in the infiltration of immune cells, such as CD8 + T cells, monocytes, and neutrophils, between the SONFH samples and the control group (Fig. 6D). Moreover, when comparing clusters C1 and C2, differences in the infiltration of CD8 + t cells, monocytes, macrophages M2 and neutrophils were also observed relative to the control group.

### WGCNA based on immune cell infiltration

**Fig. 7:**
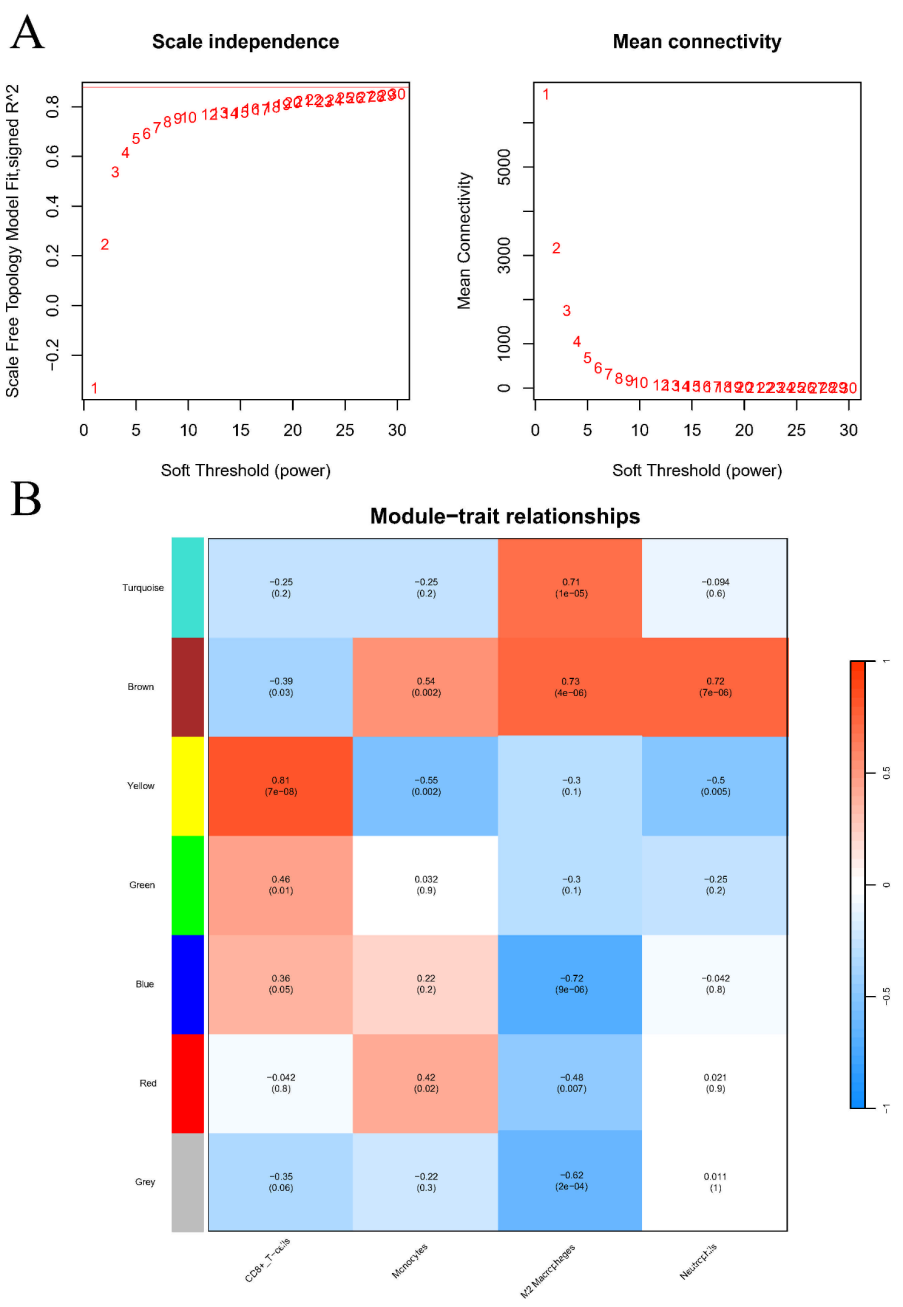
Results of WGCNA based on immune cell infiltration levels. (**A**) Selection of the optimal soft threshold. (**B**) Correlation analysis of gene modules with immune cell infiltration

To delve deeper into the association between key genes and specific immune cells, including CD8 + T cells, monocytes, macrophages M2, and neutrophils, we leveraged the results from the prior immune cell infiltration analysis as phenotypic data for a Weighted Gene Co-expression Network Analysis (WGCNA). A scale-free network was established with a β value of 18 (Fig. 7A). This analysis uncovered a significant correlation (|cor| > 0.3 and *P* < 0.05) between the genes in the brown module and the infiltration levels of the aforementioned immune cell types (Fig. 7B). From the brown module, genes that intersected with the previously identified key genes were extracted, revealing five genes specifically associated with these immune cells: CD86, ICAM1, NR3C1, IKBKB, and CD163.

#### Hub genes screening and prediction model construction

**Fig. 8:**
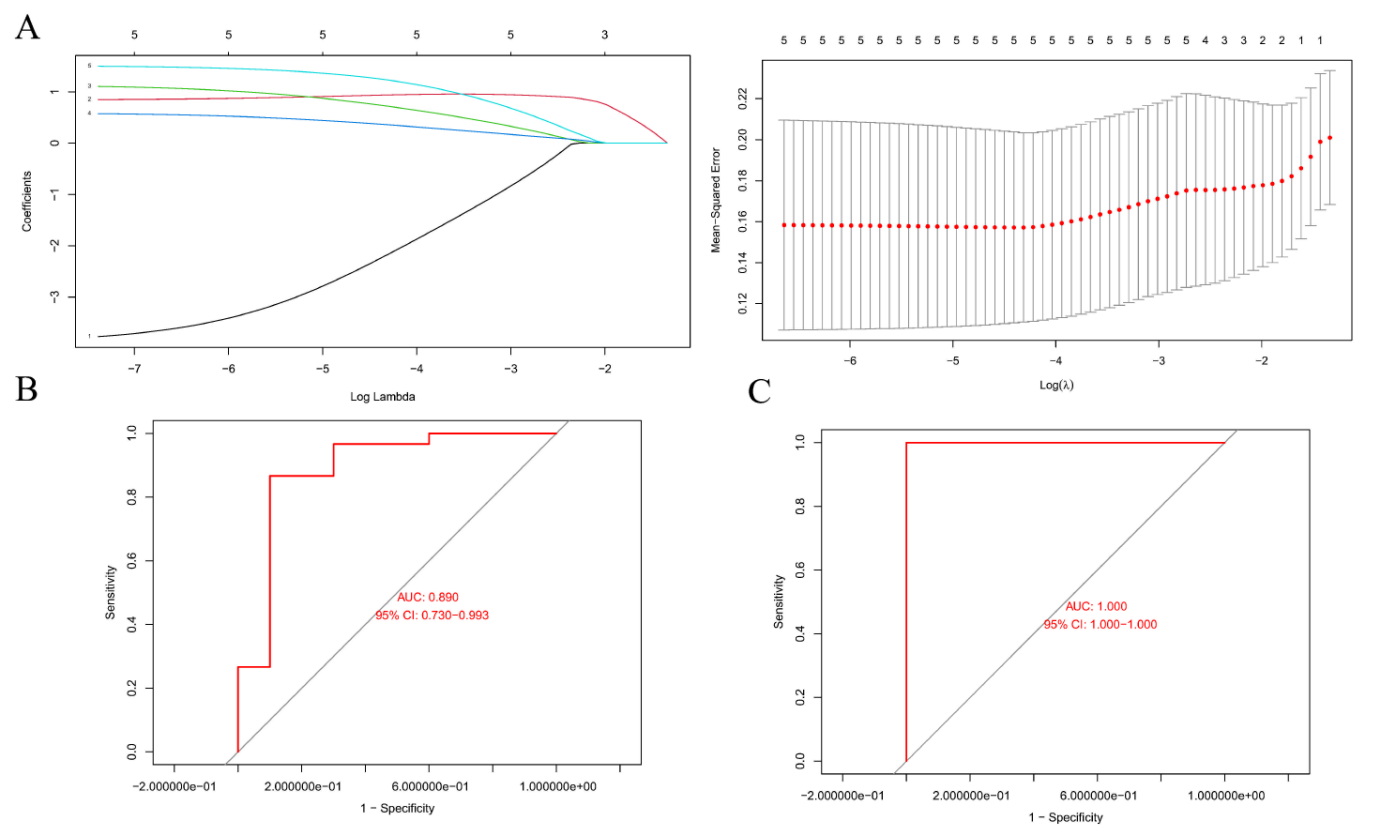
Machine learning model evaluation. (**A**) Selection of hub genes using lasso regression. (**B**) Internal validation of the model using the ROC curve. (**C**) External validation of the model using the ROC curve

Using LASSO regression and ten-fold cross-validation, three hub genes, ICAM1, NR3C1, and IKBKB, were identified (Fig. 8A). A predictive model was constructed based on these hub genes. To assess the model’s predictive performance, internal validation was performed on the GSE123568 dataset. The calculated area under the ROC curve (AUC) was 0.890 (95% CI: 0.730–0.993) (Fig. 8B). Subsequently, the predictive model was externally validated using the GSE74089 dataset to evaluate its generalizability, resulting in an AUC of 1.000 (95% CI: 1.000–1.000) (Fig. 8C), indicating that these hub genes possess excellent predictive power in both internal and external validation.

### Results of HE staining

**Fig. 9:**
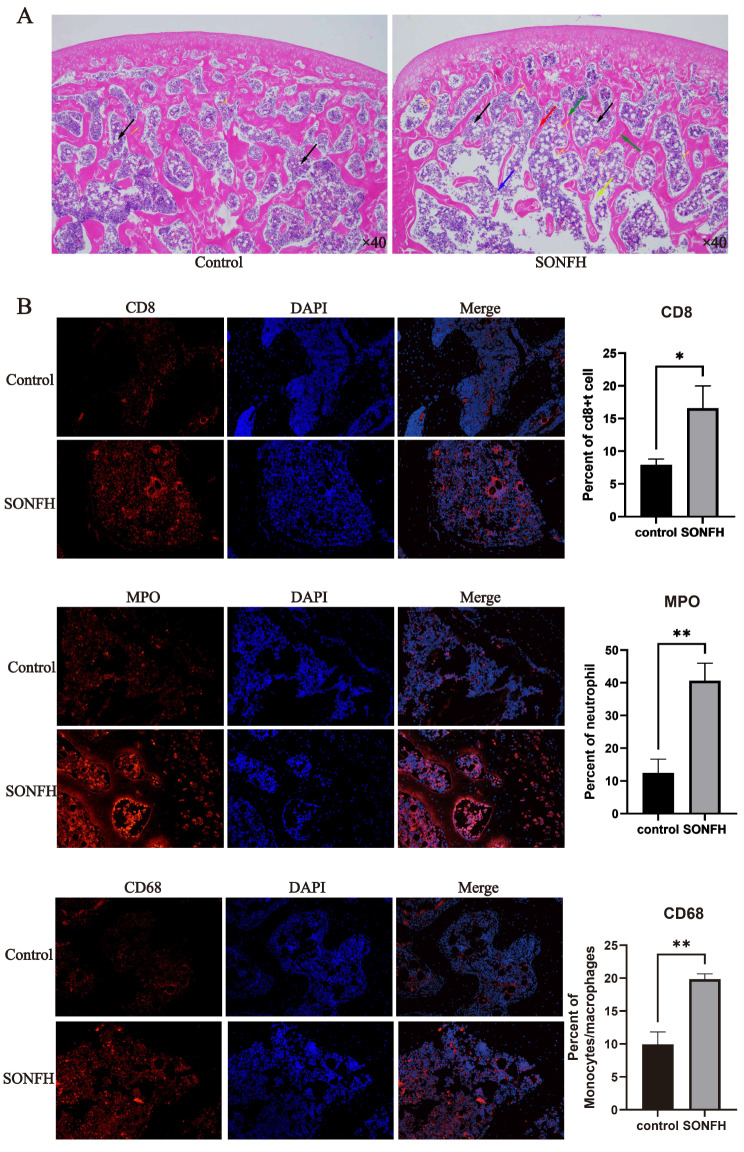
Rresults of HE staining of femoral head tissue sections and immunofluorescence detection of CD8 + t cells, neutrophils and Monocytes/macrophages. (**A**) Results of HE staining of control (left) and SONFH group (right). The SONFH group showed an obvious femoral head necrosis phenotype, characterized by bone trabecular breaks, bone marrow steatosis, andosteoclastic vacuoles, etc. (**B**) CD8 + t cells (top), neutrophils (middle) and Monocytes/macrophages (bottom) were stained with fluorescently labeled anti-CD8, anti-CD68 and anti- Myeloperoxidase for staining. Target proteins were stained red (first column), nuclei were stained blue (second column), images were me rged (third column) and results were analyzed using imagej (fourth column)

The results (Fig. 9A) in SONFH group showed irregular arrangements, thinning, and narrowing of trabeculae (red arrows), accompanied by an increased incidence of microcracks (green arrows), and fractured and missing (blue arrows). Additionally, there is an increased number of vacated osteocytic lacunae on the trabeculae (small brown arrows). In the bone marrow cavity, there is an increase in both the number and size of adipocytes (black arrows), accompanied by a reduction in bone marrow cellular content (yellow arrows).In contrast, in the control group, the bone trabeculae were structurally intact and densely arranged, with a small number of empty bone trabeculae (small brown arrowheads), and a small number of adipocytes (black arrowheads) were seen in the bone marrow cavity, with no obvious abnormality. The results showed necrosis of the femoral head in rats in the SONFH group.

#### Results of immunofluorescence

CD8, MPO (Myeloperoxidase), and CD68 are commonly recognized as markers for CD8 + T cells, neutrophils, and monocytes/macrophages. Employing the respective antibodies for immunofluorescence staining of these cells (Fig. 9B), the results indicated a significantly higher infiltration ratio of CD8 + T cells, neutrophils, and monocytes/macrophages in the SONFH group compared to the control group (*p* < 0.05).

#### Results of real-time qPCR

**Fig. 10:**
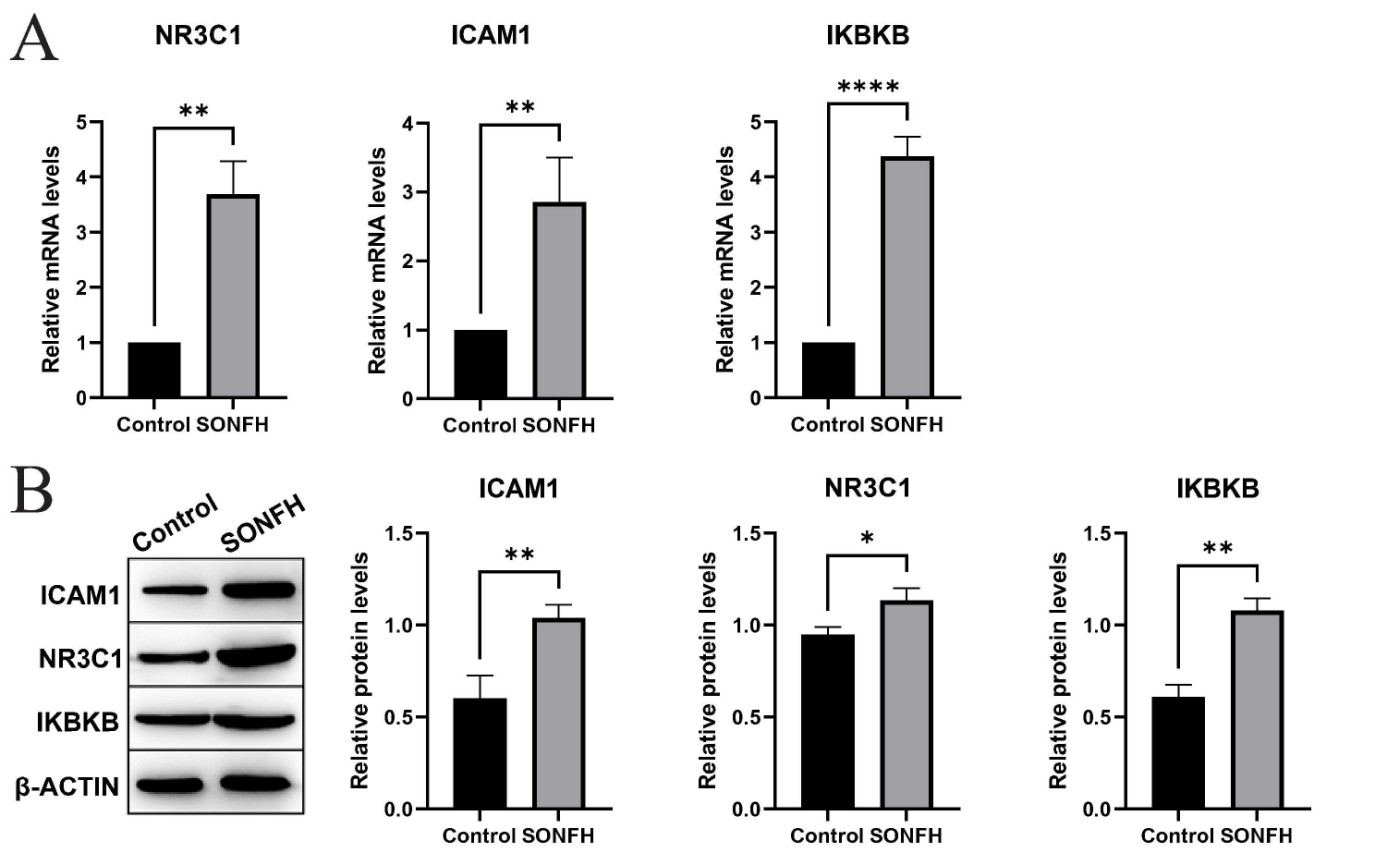
Results of qPCR and western blot (NR3C1, ICAM1,IKBKB ). (**A**) Results of qPCR. (**B**) Result of western blot. Cropped Westernblot images (left) and results analyzed using imagej (right)

We obtained rats femoral head tissue for qPCR. The results(Fig. 10A) showed that ICAM1, NR3C1 and IKBKB were significantly upregulated at the transcriptional level in the SONFH group, which may lead to femoral head destruction.

#### Results of Western blot

We analyzed the western blot results using imagej, and the results(Fig. 10B) showed that the expression of all three hub genes in the SONFH group was differentially elevated compared to the control group, and the results were consistent with qPCR. Note that Fig. 10B is a cropped image of the westernblot of the original gel presented in Supplementary Material.

## Discussion

While corticosteroids deliver substantial therapeutic advantages, their use is tempered by adverse effects such as obesity, hyperglycemia, hyperlipidemia, and osteoporosis, particularly with high-dose or long-term administration, thus constraining their clinical application [21]. Corticosteroid therapy has recently emerged as a significant risk factor for SONFH, but the pathogenic mechanisms remain elusive [22], and effective preventative treatments are lacking. This study utilized differential expression and network analyses, including WGCNA and PPI networks, to identify genes that play a pivotal role in SONFH. Enrichment analyses revealed that these key genes are predominantly involved in pathways such as Toll-like receptor signalling, lipid and atherosclerosis, and NF-kappa B signalling. Moreover, unsupervised clustering of SONFH samples underscored distinct differences in immune cell infiltration, particularly CD8 + T cells, monocytes, and neutrophils, between the control group and SONFH subgroups C1 and C2. Additional WGCNA was conducted based on immune cell infiltration to delve deeper into the immune response, pinpointing brown module genes strongly correlated with the infiltration of the aforementioned immune cells and identifying three key genes. Leveraging machine learning, the hub genes ICAM1, NR3C1, and IKBKB were ascertained and subsequently validated internally and externally, demonstrating their robust predictive capabilities. These hub genes hold potential as biomarkers for assessing immune status and facilitating early detection of SONFH.

TIAN et al. [23]. developed rat models to study the effects of methylprednisolone, establishing groups for intervention, model, and control. Through immunohistochemistry, real-time fluorescence quantitative PCR, and western blotting, they assessed the expression of TLR4, MyD88, NF-κB p65, and MCP-1 signalling molecules. The study found significant upregulation of both mRNA and protein levels of TLR4 signalling molecules in the intervention group compared to the control group. These results indicate that corticosteroids may disrupt the immune response via the TLR4 signalling pathway and contribute to the onset of femoral head osteonecrosis. This aligns with findings by S. Okazaki [24] and Shunichiro Okazaki [25], who underscored the relevance of the TLR4 signalling pathway in the pathogenesis of femoral head osteonecrosis and its augmentation by corticosteroids. Additionally, the IL-17, TNF, and NF-κB signalling pathways have been implicated in the progression of SONFH. Ren [26] reported that Th17 cells secrete IL-17 and TNF, activating signalling pathways involving TRAF, RIP1, FADD, and TAK1. RIP1 directly triggers NF-κB, while TAK1 activates p38, JNK, and NF-κB. Moreover, IL-17 interacts with IL-17R to mediate NF-κB and MAPK signalling. These downstream effectors control the transcriptional expression of AP-1, c-Fos, c-Jun, and PTGS2, elevating osteoblast activity and negatively impacting osteoclasts and endothelial cells. The disease group demonstrated increased TNF-α expression, which influences the early stages of avascular necrosis of the femoral head by driving autophagy and apoptosis of osteoblasts through the p38 MAPK/NF-κB pathway [27]. Pei et al. [28] identified that excessive activation of TLR4/NF-κB suppresses the Wnt/β-catenin pathway, leading to SONFH. However, pyrrolidine dithiocarbamate (PDTC) treatment substantially lowered NF-κB levels, reduced inflammation, and mitigated bone resorption processes such as osteolysis, adipogenesis, and apoptosis. PDTC also restored the Wnt/β-catenin pathway, promoting bone formation activities like osteogenesis and angiogenesis, thereby decreasing the incidence of SONFH. Hence, targeting the NF-κB pathway could represent a novel therapeutic approach for SONFH.

SONFH is intimately linked to the immune response. Our examination of immune cell infiltration displayed pronounced variances in the prevalence of different immune cell types, including CD8 + T cells, monocytes, macrophages M2, and neutrophils, between patients with SONFH and those in the control group. Existing research has exposed a robust association between an increase in macrophage count, the usage of glucocorticoids in individuals with SONFH, and the onset of SONFH, hinting that macrophage infiltration might play a role in the disease’s pathogenesis [29]. Tianbo et al. have suggested that polymorphisms in the interleukin-4 (IL-4) gene could relate to a predisposition to SONFH, leading to a decrease in M1 macrophages while sustaining M2 macrophage activation [30]. Furthermore, patients with SONFH have shown inflammatory infiltration of macrophages M2 and CD4 + naive T cells. Our research concurs with the observation of differential immune infiltration between the SONFH and control cohorts. Nevertheless, the precise implications of CD8 + T cell infiltration in SONFH remain to be further elucidated. Our findings also indicated elevated monocyte levels in the SONFH group compared to the control group, suggesting a possible collaborative effect of macrophages M2 and monocytes in the aetiology of SONFH, consistent with the conclusions of earlier studies. Mayu and colleagues identified the presence of neutrophil extracellular traps (NETs) within the microvasculature surrounding the femoral head in patients affected by osteonecrosis, indicating the potential involvement of neutrophils in developing SONFH [31]. A substantial body of evidence suggests that macrophages and monocytes act as progenitors to osteoclasts. Osteoclast-mediated bone resorption has been identified as a critical mechanism leading to the collapse of the femoral head [32, 33]. Consequently, administering high-dose glucocorticoids can activate and infiltrate neutrophils, monocytes, and macrophages into femoral head tissue. This infiltration likely facilitates local osteoclast differentiation, thereby disturbing the equilibrium between bone formation and resorption and contributing to the progression of SONFH.

Recent research has focused on identifying biomarkers pertinent to SONFH, among which the type I collagen cross-linked C-terminal peptide and the amino-terminal peptide of type I procollagen have been examined [34]. However, these biomarkers are also implicated in the pathogenesis of osteoporosis, which may limit their specificity and utility as diagnostic tools for SONFH [35]. This overlap underlines the necessity for new biomarkers that can more accurately aid in the prediction, diagnosis, and therapeutic management of SONFH. Our research is directed toward discovering key genes that substantially influence the immune response in SONFH, with particular emphasis on ICAM1, NR3C1, and IKBKB, which could serve as promising biomarkers for the condition.

Inhibitor of Nuclear Factor Kappa B Kinase Subunit Beta (IKBKB) is a key enzyme in the canonical IKK/NF-κB signalling pathway. This pathway’s activation involves the phosphorylation of IκB proteins by IKK, which leads to their degradation and subsequently to the release and nuclear translocation of NF-κB transcription factors. NF-κB is central to regulating various immune response genes and plays a vital role in T cells’ development, differentiation, and function. Disruption of IKBKB function can result in diminished T cell populations, developmental abnormalities, and dysfunctional immune responses. IKBKB also has a critical function in B cell maturation, immune response, survival, and the production of antigen-specific antibodies. Additionally, its involvement extends to antigen presentation and immune responses in dendritic cells and macrophages, underscoring its extensive impact on the immune system. The importance of IKBKB in these processes is becoming increasingly recognized [36]. Liu [37] found that patients with SONFH have reduced levels of the inhibitor of nuclear factor kappa-B kinase epsilon (IKKe), which interacts with IKBKB in the NF-κB signalling pathway. IKKe can suppress IKBKB by inhibiting its transcription, leading to decreased osteoblast activity. The knockdown of IKKe enhances osteoclastogenesis in mice and dampens the pro-inflammatory response in a SONFH mouse model, suggesting a complex interplay in bone homeostasis. Nuclear Receptor Subfamily 3 Group C Member 1 (NR3C1) is essential for mediating inflammatory responses, cell proliferation, and differentiation in target tissues. Mutations in NR3C1 can lead to glucocorticoid resistance. In mice with osteoblast-specific NR3C1 deletion, there is a significant decrease in trabecular bone mass in the vertebrae, along with suppressed expression of osteogenic genes in osteoblasts [38]. ICAM1, on the other hand, is an integral component of bone metabolism, especially in osteoclastogenesis. It is expressed on osteoblasts in response to various stimuli, including stress, and enhances adhesion, which is crucial for osteoclast formation and function. While ICAM1 has been associated with tumour-induced bone metastasis, its potential involvement in the pathogenesis of SONFH warrants further exploration [39].

In this study, a combination of differential gene expression analysis, WGCNA, and PPI networks were utilized to identify a set of 15 key genes associated with SONFH. Enrichment analyses were conducted on these genes to gain a deeper understanding of the molecular mechanisms involved in SONFH. Additionally, from an immunological standpoint, 5 key immune-related targets were identified, and 3 hub genes were pinpointed by applying machine learning techniques. A predictive model constructed from these hub genes demonstrated exceptional predictive accuracy, verified by internal and external validation. Animal experiments further confirmed the differences in the infiltration of hub genes and associated immune cells in SONFH and control groups. Collectively, the results of this research provide new insights into the pathogenesis of SONFH and may aid in identifying therapeutic targets for this condition.

### Electronic supplementary material

Below is the link to the electronic supplementary material.

Supplementary Material 1

## Data Availability

The datasets generated and/or analysed during the current study are available in the GEO repository (www.ncbi.nlm.nih.gov/geo/). GSE123568 is available at: https://www.ncbi.nlm.nih.gov/geo/query/acc.cgi?acc=GSE123568. GSE74089 is available at: https://www.ncbi.nlm.nih.gov/geo/query/acc.cgi?acc=GSE74089

## Abbreviations

SONFH: Steroid-induced osteonecrosis of femoral head
WGCNA: Weighted gene co-expression network analysis
PPI: Protein-protein interaction
COVID-19: The novel coronavirus pandemic
IgG: Immunoglobulin G
GEO: Gene Expression Omnibus database
HE: Hematoxylin-eosin
EDTA: Ethylenediamine tetraacetic acid
PVDF: Polyvinylidene difluoride
SDS-PAGE: SDS-polyacrylamide
DEGs: Differentially expressed genes
PDTC: Pyrrolidine methiocarb carbamate
IL-4: Interleukin-4
IKBKB: Kappa B Kinase Subunit Beta
IKKe: Kappa-B kinase epsilon
NR3C1: Nuclear Receptor Subfamily 3 Group C Member 1
