# Supplementary material for: Identification of steroid-induced osteonecrosis of the femoral head biomarkers based on immunization and animal experiments
Source: BMC Musculoskelet Disord. 2024 Jul 29;25:596. doi: 10.1186/s12891-024-07707-4 (PMC11285486; doi:10.1186/s12891-024-07707-4)
Supplement: Supplementary file 1 — Supplementary Material 1 [file 12891_2024_7707_MOESM1_ESM.pdf]

HE staining

Control

SONFH

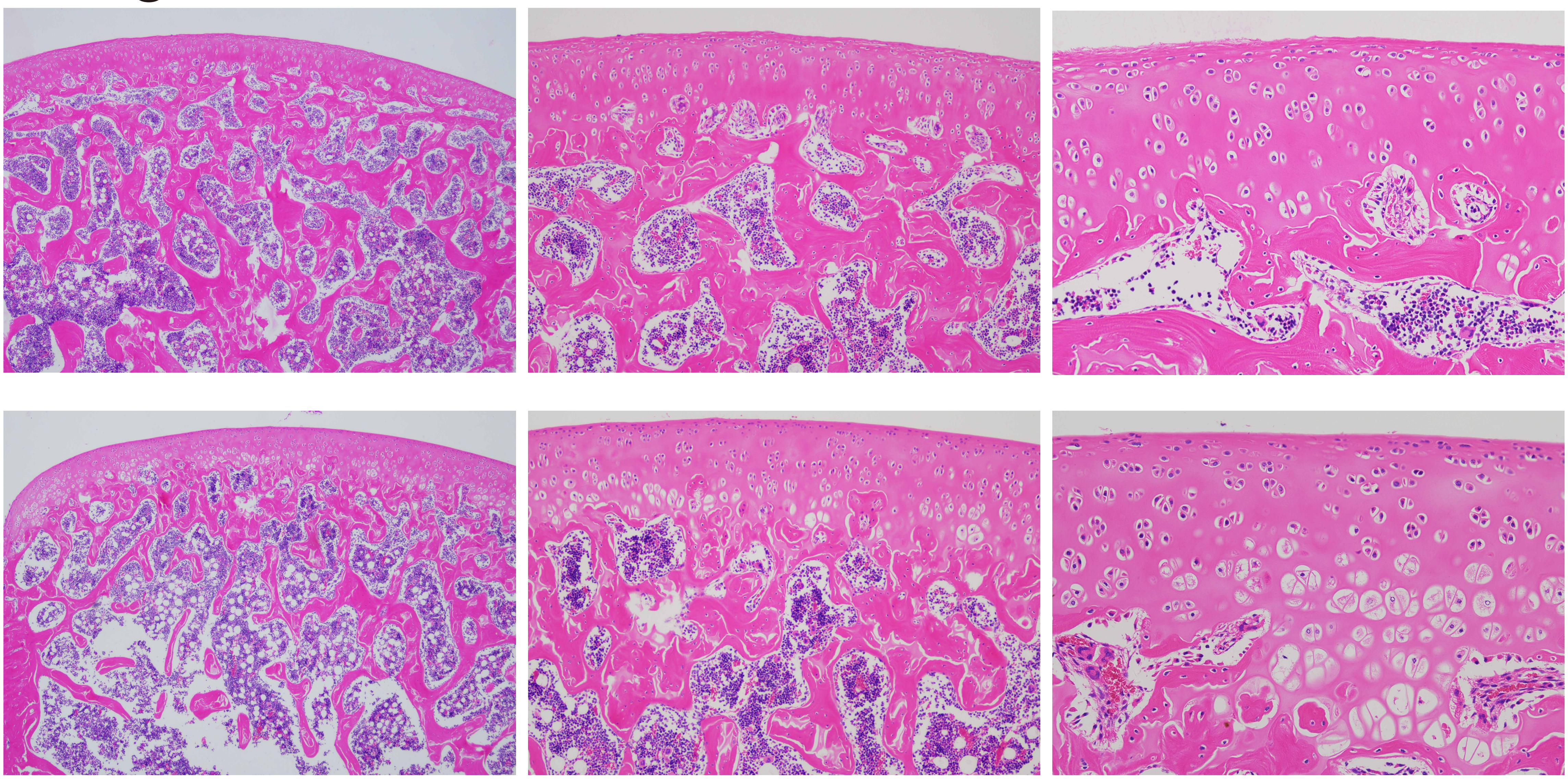

Western blot

ICAM1

NR3C1

IKBKB

β-ACTIN

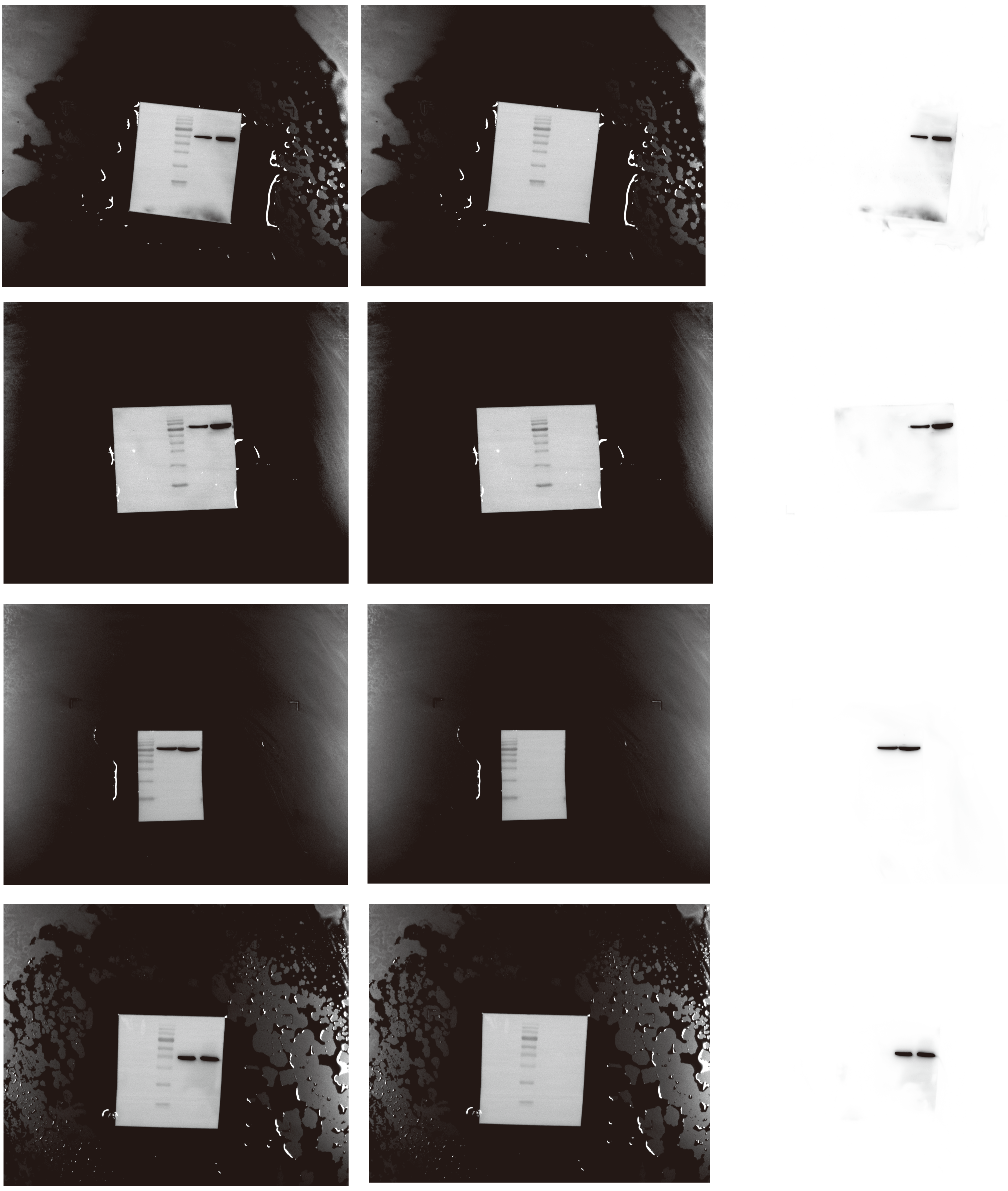

qPCR

primer	"sequences（5’ to 3’）"
Rat actin F	CCCGCGAGTACAACCTTCTT
Rat actin R	AACACAGCCTGGATGGCTAC
Rat ICAM1 F	TTCCCTGGAAGGCCTGTTTC
Rat ICAM1 R	GGGAAGTACCCTGTGAGGTG
Rat IKBKB F	CGGGAGAATGACGTGAAGGT
Rat IKBKB R	AGCAGCAGCCGTACCATATC
Rat NR3C1 F	AAGAGCAGTGGAAGGACAGC
Rat NR3C1 R	TGGTATCGCCTTTGCCCATT
